# Psychometric evaluation of the traditional Chinese version of the resilience Scale-14 and assessment of resilience in Hong Kong adolescents

**DOI:** 10.1186/s12955-020-01285-4

**Published:** 2020-02-19

**Authors:** Joyce Oi Kwan Chung, Katherine Ka Wai Lam, Ka Yan Ho, Ankie Tan Cheung, Long Kwan Ho, Viveka Wei Xei, Faith Gibson, William Ho Cheung Li

**Affiliations:** 1 School of Nursing, University of Hong Kong, 4/F, William M. W. Mong Block, 21 Sassoon Road, Pokfulam, Hong Kong; 2 School of Health Sciences, University of Surrey, Guildford, UK

**Keywords:** Adolescent, Depressive symptoms, Psychometrics, Resilience, Self esteem

## Abstract

**Background:**

A reliable and valid instrument that accurately measures resilience is crucial for the development of interventions to enhance the resilience of adolescents and promote their positive mental well-being. However, there is a lack of adolescent resilience assessment tools with good psychometric properties suitable for use with Hong Kong participants. This study aimed to evaluate the psychometric properties of the traditional Chinese version of the Resilience Scale-14.

**Methods:**

Between October 2017 and January 2018, a stratified random sample of 1816 Grade 7 (aged 11–15 years) students from all 18 districts of Hong Kong were invited to participate in the study. Subjects were asked to respond to the traditional Chinese version of the Resilience Scale-14, the Center for Epidemiologic Studies Depression Scale for children, and Rosenberg’s Self-Esteem Scale. The psychometric properties, including the internal consistency, content validity, convergent and discriminant validity, exploratory and confirmatory factor analyses, and test–retest reliability of the Resilience Scale-14 were assessed.

**Results:**

The translated scale demonstrated good internal consistency and test–retest reliability, excellent content validity, and appropriate convergent and discriminant validity. The results of the confirmatory factor analysis supported the two-factor structure of the traditional Chinese version of the Resilience Scale-14.

**Conclusions:**

Results suggest that the translated scale is a reliable and valid tool to assess the resilience of young Hong Kong Chinese adolescents. Healthcare professionals could use the newly translated scale to assess resilience levels among Hong Kong adolescents and develop interventions that can help them combat mental health problems and lead healthier lives.

**Trial registration:**

Clinicaltrials.gov ID NCT03538145 (retrospectively registered on May 15, 2018).

## Background

The rising incidence of mental health problems in adolescents, such as emotional disturbance, adjustment and eating problems, depression and suicidal tendencies has become a major global public health concern [1–3] and are becoming more prevalent in Hong Kong [4].

Resilience is defined as an individual’s ability to utilize a range of protective factors, such as personal and social resources and perceived level of family cohesion, to maintain mental well-being in the face of stress and adversity [5, 6]. Resilience effectively prevents the development of mental health problems and is associated in adolescents with positive mental health outcomes, such as reduced levels of anxiety, depression, and obsessive–compulsive symptoms [7–9]. The assessment of resilience in adolescents is therefore crucial to develop a thorough understanding of their responses to stress and adversity. In addition, it is of paramount importance for healthcare professionals to develop and evaluate appropriate interventions that can enhance the resilience of adolescents and foster the development of their coping mechanisms and positive mental well-being. Before any interventions can be planned or evaluated, a reliable and valid instrument that accurately measures resilience in adolescents must be developed. Unfortunately, there is a lack of adolescent resilience assessment tools with effective psychometric properties suitable for the Hong Kong Chinese context.

There are various instruments to assess resilience [10]. One promising resilience scale is the Connor–Davidson Resilience Scale (CD-RISC) [11]. The 25-item CD-RISC has been used in studies in primary care, and with the general population and psychiatric outpatients, and has good validity and reliability [12]. The CD-RISC was originally developed to measure resilience in adults, and has been used to assess resilience in adolescents from mainland China [13]. Owing to the complexity of the scale’s content and the limited test-taking abilities of young adolescents, the appropriateness of the CD-RISC to assess resilience in adolescents is unclear. Another well-established resilience scale is the Resilience Scale (RS) developed by Wagnild and Young [14]. The RS, which has been translated into and validated in a variety of languages, comprises 25 items and has been widely used by researchers and healthcare professionals with various populations [11, 15, 16]. The RS is particularly appropriate for studying resilience in community samples because of its psychometric properties and applicability to a variety of age groups [11], whereas the CD-RISC is mainly used to quantify resilience in clinical settings to evaluate treatment responses [12].

Following the validation of the RS, a 14-item version, Resilience Scale-14 (RS-14) was developed [14, 17]. The RS-14 was derived from the original 25-item RS and constructed at a 4.9 Flesch–Kincaid reading level (1 year level lower than the 6th grade reading level of the RS) to facilitate comprehension and achieve appropriateness for adolescents [10]. The RS-14 has been widely used in resilience research and has been translated into and validated in a variety of languages, such as simplified and traditional Chinese for mainland and Taiwanese Chinese participants, respectively [18–21]. However, there are linguistic differences between traditional and simplified Chinese [22]. The simplified Chinese version of the RS-14 is not appropriate for use in the Hong Kong Chinese context. Although traditional Chinese characters are currently used in Taiwan, it may be psychometrically inappropriate and even problematic to apply the translated tool to a new cultural group, such as Hong Kong Chinese adolescents. Owing to cultural differences, some concepts or items in the original instrument may be inappropriate for people from other cultures [23] and it may thus yield inaccurate results [24]. Given these issues, before using a translated version of the scale in the Hong Kong Chinese context, it is crucial to evaluate its linguistic and cultural equivalence. The psychometric properties of the Chinese version of the RS-14 require further empirical testing. Confirmatory factor analysis (CFA), which can be used to test a hypothesized configuration of the factor structure or measurement model of a scale, has not been performed on the traditional Chinese version of the RS-14. The study aim was to translate the original RS-14 (English version) into traditional Chinese. The psychometric properties of the newly translated RS-14 were then empirically tested.

## Methods

This study was approved by the Institutional Review Board of the University of Hong Kong and Hospital Authority Hong Kong West Cluster (reference UW17–378). The principal and teachers of each school were fully informed about the study’s purpose, nature, design, and duration. In addition, parents were sent an information sheet and a consent form via the schools to inform them that a study was to be conducted to examine issues relevant to adolescent health. Parents were given the option to participate or to refuse to let their child be involved in the study by returning the signed consent forms. In addition, verbal consent was obtained from all individual subjects and they were given the option to participate or to decline to participate in the study.

### Design and participants

A test–retest, within-subjects design was used and the data were collected between October 2017 and January 2018.

There are no clear guidelines as to sample size for factor analysis, and there is little agreement among researchers regarding how large a sample should be. Although there is no evidence to support the rule of “the larger, the better,” most researchers suggest using a larger sample [25, 26]. Gorsuch [27] claims that at least 200 subjects for each factor analysis is recommended. Other than the basic sample size requirement, we also aimed to survey a large and representative sample of Hong Kong Chinese adolescents. With all this in mind, a stratified random sample of Form 1 students (Grade 7) from 18 secondary schools across 18 districts in Hong Kong were invited to participate in the study. Students at these schools were randomly selected and invited to participate in the proposed study. A serial code was assigned to every secondary school in the identified districts according to its alphabetic order. By using the serial codes, a personal computer program then randomly selected one school from each district. This procedure was conducted by a research assistant, which is blinded to the researchers. An invitation letter describing the nature and purpose of the study was sent to the identified secondary schools. If a selected school refused to participate, the computer program would randomly select another school from the same district.

A total of 1837 parents of adolescents from 18 schools were sent an information sheet and a consent form via the schools between September 2017 and January 2018. However, 16 parents from 9 schools did not return the consent form before the deadline. The response rate is 99.1%. The remaining 1821 parents who signed the consent forms and agreed their child to participate the study. All invited adolescents were able to speak Cantonese and read Chinese and no one had identified cognitive and learning problems. However, we subsequently received five largely incomplete questionnaires. Therefore, 1816 questionnaires from a total eligible pool of 1821 students were used for the analysis.

### Measures

#### Resilience Scale-14

The RS-14 is a 14-item scale that measures two factors: personal competence, and acceptance of self and life. Each item is answered using a 7-point Likert scale ranging from ‘strongly disagree’ to ‘strongly agree’, with total possible scores ranging from 14 to 98. Higher scores indicate higher levels of resilience.

#### The Center for Epidemiologic Studies Depression Scale for children (CES-DC)

Depressive symptoms were assessed with the Chinese version of the CES-DC. The CES-DC comprises 20 fully standardized items to evaluate depressive symptoms. All items are evaluated on a 4-point self-report scale in relation to their incidence during the previous week, and scored from 0 to 3. Total possible scores range from 0 to 60; higher scores indicate greater symptomatology.

The psychometric properties of the Chinese version of the CES-DC have been empirically tested. The scale shows adequate internal consistency reliability (*r* = 0.82), good content validity (content validity index [CVI] = 95%), and appropriate convergent (*r* = 0.63) and discriminant (*r* = − 0.52) validity [4].

#### Rosenberg’s self-esteem scale (RSES)

Self-esteem was assessed with the Chinese version of the RSES. The RSES is designed to measure global self-esteem in children and adolescents. It comprises 10 items rated on a 4-point Likert scale ranging from 1 to 4; total possible scores range from 10 to 40. Higher scores indicate higher levels of self-esteem.

The Chinese version of the RSES has previously been used with children [28] and adolescents [3]. Findings demonstrate adequate internal consistency reliability (*r* = 0.84) and appropriate discriminant validity (*r* = − 0.52).

#### Issues related to instrument translation

The RS-14 was translated and back-translated following the World Health Organization guidelines on the process of translation and adaptation of instruments (http://www.who.int/substance_abuse/research_tools/translation/en/) and following the technique described by Bracken and Barona [29]. The 14 items of the RS-14 were first translated from English to traditional Chinese by the researcher (JOKC). Another translator, blinded to the original items, completed the back-translation. Conceptual rather than literal meaning was the aim in translation. The retranslated English version and the original English version were then compared to check if the meaning of each item had been maintained. Discrepancies were discussed and agreed upon by both the researcher and the back-translator.

### Data collection

All subjects were asked to complete the Chinese version of the RS-14, CES-DC, and RSES by themselves on the day of recruitment at their schools. To examine the test re-test reliability, a total of 426 students from six secondary schools (randomly selected from 18 districts) were invited (with parental consent) to complete the RS-14 again after 2 weeks at their schools. All the questionnaires were distributed and collected by a research assistant After filling in the questionnaires, all subjects were given an information pamphlet about mental well-being (Chinese version) published by the Centre for Health Protection of the Department of Health in Hong Kong. Hotline numbers for professional counselling on mental well-being were printed inside the information pamphlet. Subjects were informed that they could call the hotline for counselling if they needed to.

### Data analysis

#### Semantic and content equivalence

The newly translated Chinese version of the RS-14 was subjected to equivalence testing of its semantic and content dimensions. A panel of experts was set up to examine the semantic and content equivalence of the newly translated Chinese version of the RS-14. The panel included the researcher, an associate professor with rich experience in conducting research on children and adolescents, a child clinical psychologist, a biostatistician, and two lecturers with experience in teaching mental well-being for adolescents. All the experts were bilingual and experienced in translation and validation of instruments.

#### Semantic equivalence

Using a 4-point rating scale (from 1 = not equivalent to 4 = most equivalent), the panel of experts was asked to rate the equivalence of translation between each item of the original English and Chinese versions of the RS-14. An equivalence rate (the percentage of the total items rated by the experts as either 3 or 4) was then calculated. Any item deemed not equivalent (i.e. a rating of 1 or 2) by more than 20% of respondents was amended.

#### Content equivalence

Using a 4-point rating scale (from 1 = not relevant to 4 = very relevant), the panel of experts was asked to rate the content equivalence of the Chinese version of the RS-14. The CVI is the percentage of the total items rated as either 3 or 4. A CVI score of 80% or higher is generally considered to indicate good content validity [30].

#### Construct validity: internal (factorial structure)

To examine the underlying factor structure of the traditional Chinese version of RS-14, exploratory factor analysis (EFA) was first performed and then followed by CFA to evaluate whether the proposed factor structure by EFA might adequately fit the data. As CFA would need to be performed on a different set of data to confirm the results of an EFA [31], the original data set (*N* = 1816) was randomly split into two (dataset A & B). EFA was performed on the dataset A (*N* = 908) and CFA was performed on the dataset B (*N* = 908).

To examine the factorial structure of the Chinese version of the RS-14, EFA was performed using the Statistical Package for Social Sciences (SPSS) software, version 23.0 for Windows (SPSS Inc., Chicago, IL, USA). Prior to performing EFA, the suitability of the data set for factor analysis was confirmed using Bartlett’s test of sphericity and the Kaiser–Meyer–Olkin measure of sampling adequacy. A principal components analysis was used. Two techniques of factor extraction, Kaiser’s criterion and Cattell’s [32] scree test, were used to help determine the number of factors to be retained for further investigation. With reference to Kaiser’s criterion, only factors with an eigenvalue of 1 or above are retained for further investigation. For the scree test, Cattell [32] recommended that all factors above the elbow, or break in the plot, should be retained, as these factors explain most of the variance in the data set. As recommended by Watson and Thompson [33], both orthogonal and oblique rotation methods were used.

CFA was carried out using LISREL version 8.8 for Windows (Scientific Software International Inc., Lincolnwood, IL, USA). The parameters were estimated using the generally weighted least squares method, using asymptotic covariance matrix. The overall fit of the data model with the scale was then examined using goodness of fit indices, including the chi-square/degrees of freedom ratio (χ^2^/d.f. ratio), root mean square error of approximation (RMSEA), comparative fit index (CFI), and Tucker-Lewis index (TLI). The χ^2^/d.f. ratio is a measure of global fit. A χ^2^/d.f. value between 1 and 5 indicates good fit [34].

#### Construct validity: external (relationships with external measures)

##### Convergent and discriminant validity testing

Prior to performing correlational analyses for convergent/discriminant validity, preliminary assumption testing was conducted to check for normality. By an inspection of the histograms and the normal probability plots (Normal Q-Q Plots) the data obtained were found to be normally distributed.

There are two factors in the RS-14: personal competence, and acceptance of self and life. Convergent validity was established by showing how strongly correlated among items within personal competence and acceptance of self and life. Whereas, discriminant validity was demonstrated by showing how the personal competence and acceptance of self and life were less correlated.

Construct validity (external) was further established by examining the correlation between scores on the Chinese version of the RS-14 and CES-DC scores, and that between scores on the Chinese versions of the RS-14 and RSES using the Pearson product-moment correlation coefficient.

##### Reliability testing

Internal consistency reliability of the Chinese version of the RS-14 was assessed by calculating Cronbach’s alpha. To examine the stability of the RS-14, 426 subjects were asked to complete the scale again after 2 weeks. The intraclass correlation coefficient (ICC-consistency) was used to estimate the test–retest reliability coefficient.

## Results

The participant demographic characteristics are shown in Table 1. The data indicate that there were similar numbers of boys and girls. The age ranged from 11 to 15 years. Around 15% of students came from single parent families. We found that participants were able to provide full responses to the questionnaires, without showing any particular difficulty in understanding the questions. It took around 10 to 15 min for each adolescent to fill in all questionnaires.

**Table 1:** Demographic Characteristics of the Participants (*N* = 1816)

	Frequency	%
Age (Yrs)		
11	191	10.5%
12	1181	65.0%
13	417	23.0%
14	22	1.2%
15	5	0.3%
Sex		
Male	878	48.3%
Female	938	51.7%
Parental marital status		
Live with both parents	1542	84.9%
Single parent family	274	15.1%
Parents’ Educational Attainment		
Primary school or below	108	5.9%
Lower secondary school	472	26.0%
Upper secondary school	865	47.7%
Tertiary education	371	20.4%

### Semantic and content equivalence

To achieve semantic equivalence, each item must remain idiomatically and conceptually the same after translation; to achieve content equivalence, each item should be culturally relevant [24].

### Semantic equivalence

The equivalence rate was 97%, indicating that each item of the Chinese version of the RS-14 remained idiomatically and conceptually the same as in the English version.

### Content equivalence

The CVI was 95%, indicating that the content of the Chinese version of the RS-14 was valid.

### Construct validity: internal (factorial structure)

#### Exploratory factor analysis

Principal components analysis revealed the presence of two components with eigenvalues exceeding 1, which explained 41.95 and 8.61% of the variance, respectively.

An inspection of the scree plot revealed a clear break after the second component. Therefore, it was decided to retain two components for further investigation. To aid in the interpretation of these two components, both orthogonal and oblique rotation methods were used. Both methods produced similar derived factor analytic solutions. However, the oblique rotated solution generated by the direct oblimin procedure revealed the presence of a simple structure [35], which was easier to interpret. Therefore, the result of the oblique rotation was reported in the present study (Table 2). The two-factor solution explained 50.56% of the total variance. The interpretation of the two components was consistent with the proposed factor structures of the original RS-14 (English version).

**Table 2:** Two-factor solution for the Chinese version of the Resilience Scale-14

Items	Component 1 Personal competence	Component 2 Acceptance of self and life
I usually manage one way or another	.598	
I feel proud that I have accomplished things in life	.618	
I feel that I can handle many things at a time	.694	
I am determined	.697	
I can get through difficult times because I’ve experienced difficulty before	.688	
I have self-discipline	.657	
I keep interested in things	.571	.340
My belief in myself gets me through hard times	.539	
In an emergency, I’m someone people can generally rely on	.437	.341
When I’m in a difficult situation, I can usually find my way out of it	.669	
I usually take things in stride		.563
I am friends with myself		.768
I can usually find something to laugh about		.764
My life has meaning		.801
% of variance explained	27.92	22.64

Note: Only loadings above .3 are reported

#### Confirmatory factor analysis

**Fig. 1:**
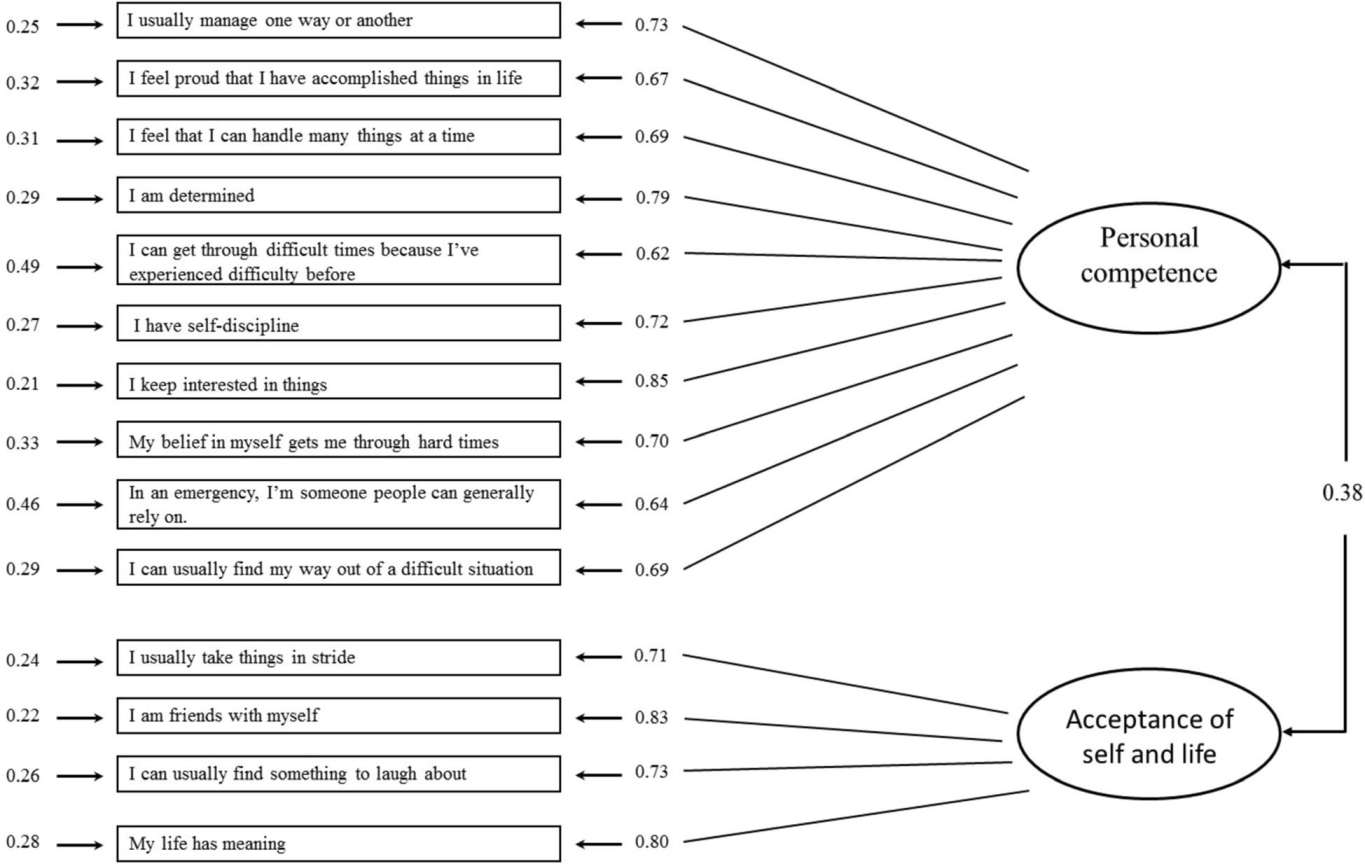
Confirmatory factor analysis model for the traditional Chinese version of the Resilience Scale-14

Fig. 1 shows the parameter estimates of this two-factor model. All correlation matrices were less than 1 and were positive definite, indicating that the parameter estimated was reasonable. The factor loading for each observed variable was high, ranging from 0.62 to 0.85. The *t*-values of all variables were greater than 2.00, suggesting statistically significant loadings. The standard errors ranged from 0.21 to 0.49, indicating that all the parameters were accurately estimated [36]. The results of the goodness of fit indices, including the χ^2^/d.f. ratio, RMSEA, CFI and TLI were 3.37, .05, .96 and .96, respectively, indicating a good model-data fit.

##### Construct validity: external (relationships with external measures)

Following Cohen [37], correlation coefficients of .10 to .29, .30 to .49, and .50 to 1.0 were interpreted as indicating small, medium, and large effects, respectively. There was a moderate positive correlation between scores on the Chinese version of the RS-14 and RSES scores (*r* = 0.38, *n* = 1816, *p* < 0.01), indicating that adolescents with higher resilience also reported higher levels of self-esteem. In addition, there was a strong negative correlation between RS-14 and CES-DC scores (*r* = − 0.50, *n* = 1816, *p* < 0.01), indicating that greater resilience in adolescents was associated with fewer self-reported depressive symptoms.

#### Convergent and discriminant validity

The correlation coefficients among items within personal competence and acceptance of self and life ranged from 0.62–0.85 and 0.71–0.83, respectively. This showed that the items of each factors in the RS-14 were strongly correlated. In addition, there was a moderate negative correlation between personal competence and acceptance of self and life (*r* = 0.38, *n* = 1816, *p* < 0.01), indicating that these two factors are less correlated when compared to the items within the same factors.

#### Reliability

The alpha coefficients for the internal consistency of the Chinese version of the RS-14 was 0.86. High item–total correlations, ranging from 0.521 to 0.77, were also found for responses to most items on the Chinese version of the RS-14. The test–retest reliability coefficient at the 2-week interval was 0.84.

## Discussion

In this study, multiple investigations were carried out to assess the adequacy of validity of the RS-14. These included both EFA and CFA, and findings correlations with other instruments which were intended to measure the same (convergent validity) or different constructs (discriminant validity). All these investigations were consistent with the benchmark proposed by the EFPA 2013 revised Test Review Model (http://www.efpa.eu/professional-development/assessment) that provides descriptions on rigorous assessment of psychometric properties. Hence, the results of this study adequately reflect the validity of the RS-14.

The overall results of this study showed that the traditional Chinese version of the Resilience Scale-14 demonstrated good internal consistency and test–retest reliability, excellent content validity, and appropriate convergent and discriminant validity. The confirmatory factor analysis supported the two-factor structure of the traditional Chinese version of the Resilience Scale-14.

There were several reasons for inviting Form 1 students to participate in the study. The move from primary to secondary school can be a very stressful experience, which may create a potential threat to adolescents [38]. This may be compounded by changes in academic and social expectations that render children more psychologically vulnerable. In addition, adolescents of this age are in a stage of complex transition. According to social development theory [39], they have entered the stage of ‘fidelity’, which is dominated by role confusion, the search for a personal identity, and the influence of peers.

Consistent with a previous study on the RS-14 [20], the results of this study showed that the internal consistency of the traditional Chinese version of the RS-14 was high. The item–total correlations indicated that all items were highly correlated with the total scores. The findings suggest that these items are relatively homogenous and measure the same psychological construct, and provide empirical evidence of the reliability of the RS-14. The test–retest reliability of the newly translated instrument was also high (0.84) as estimated by the ICC-consistency. These findings are in accord with those of a previous study [20] showing that the RS-14 has good stability in measuring resilience in adolescents.

A previous study indicating that adolescents with greater resilience have higher self-esteem [40]. We hypothesized that there would be a positive correlation between scores on the Chinese version of the RS-14 and RSES scores. Our results revealed a moderate positive correlation between scores on the traditional Chinese version of the RS-14 and RSES scores. The findings indicated that the newly developed scale showed construct validity.

There is some evidence that resilience is negatively related to depressive symptoms [7, 8, 41]. We hypothesized that there would be a negative correlation between the Chinese version of the RS-14 and the CES-DC. Our results revealed a strong, negative correlation between scores on the traditional Chinese version of the RS-14 and CES-DC scores. This result provided additional evidence that traditional Chinese version of the RS-14 showed construct validity.

Our results showed that items within the same factors (personal competence, and acceptance of self and life) of the RS-14 were strongly correlated, whereas the two factors were less correlated. Hence, the newly developed scale demonstrated convergent and discriminant validity.

The EFA results provided strong evidence that there were two factors, personal competence and acceptance of self and life, underlying the traditional Chinese RS-14 structure. The interpretation of the two components was consistent with a previous factor analytic study on the simplified Chinese version of the RS-14 [20]. The two-factor solution explained 50.56% of the total variance, which was higher than the criterion of 50% of the total variance explained suggested by Streiner [42].

Although the RS-14 has been widely used in research, CFA (which can be used to test a hypothesized configuration of the factor structure of the scale) had not been performed on this scale. To allow more precise testing of the instrument’s factor structure, CFA was performed in this study to evaluate whether the factor models indicated by the EFA could adequately fit the data. The RMSEA is an indication of model fit and is based on the population discrepancy function, which is a standardized measure of error of approximation [43]. MacCallum [44] recommends that researchers should consider using RMSEA as it is an important measure of lack of fit per degree of freedom. In general, RMSEA values of less than 0.05 indicate superior model fit, although Browne and Cudeck [44] argue that RMSEA values of up 0.08 suggest a reasonable fit of the model to the population. The CFI is an indicator of how much better the model fits compared with an independence model. The TLI analyses the discrepancy between the chi-squared values of the hypothesized model, which was built on an index formed by Tucker and Lewis [45]. These measures vary from 0 to 1; a value of 0.95 or higher indicates a good fit [46]. The generally weighted least squares suggested by Jöreskog and Sörbom [47] was used for CFA parameter estimation. The results of CFA supported the two-factor structure of the RS-14.

### Limitations

The use of convenience sampling and the fact that only young adolescents (Grade 7) were recruited for the study limit the generalizability of the results. Another limitation is that only relatively healthy adolescents were recruited. It is uncertain, therefore, whether the RS-14 can differentiate groups who are known to have different characteristics. It would be interesting in the future to examine whether there is any difference in resilience between healthy adolescents and those with chronic illness.

### Implications for practice

This study addressed a gap in the literature by testing the psychometric properties of the traditional Chinese version of the RS-14 and confirmed that the scale can be used in the Hong Kong Chinese population. Healthcare professionals could use the newly translated RS-14 to assess resilience levels among Hong Kong Chinese adolescents. The newly developed RS-14 is also an appropriate clinical research tool for evaluating the effectiveness of nursing interventions and for use in other studies involving adolescents. Most importantly, healthcare professionals should collaborate more with the education sector and school social workers to develop appropriate psychological interventions that can enhance the resilience of adolescents and foster the development of their coping mechanisms and positive mental well-being. This would help adolescents to better combat mental health problems and lead healthier lives.

## Conclusions

Despite some limitations, this study provides further evidence of the factor structure of the traditional Chinese version of the RS-14. The results suggest that this scale is a reliable and valid tool to assess the resilience of young Hong Kong Chinese adolescents.

## Data Availability

The datasets analyzed during the current study are available from the corresponding author on reasonable request.

## Abbreviations

CD-RISC: Connor-davidson resilience scale
CESDC: The center for epidemiologic studies depression scale for children
CFA: Confirmatory factor analysis
CFI: Comparative fit index
CVI: Content validity index
EFA: Exploratory factor analysis
ICC: Intraclass correlation coefficient
Normal Q-Q Plots: Normal probability plots
RMSEA: Root mean square error of approximation
RS: Resilience scale
RS-14: Resilience scale-14
RSES: Rosenberg’s self-esteem scale
TLI: Tucker-lewis index
χ^2^/d.f. ratio: Chi-square/degrees of freedom ratio
